# Limited transmission of SARS-CoV-2 in schools in Ireland during the 2020–2021 school year

**DOI:** 10.2807/1560-7917.ES.2023.28.15.2200554

**Published:** 2023-04-13

**Authors:** Ciara Kelly, Philippa White, Elizabeth Kennedy, Dearbhail O’Flynn, Aoife Colgan, Mary Ward, Margaret B O’Sullivan, Claire M Buckley, Breda Cosgrove, Melissa Canny, Katharine Harkin, Fiona McGuire, Catherine Lynch, Aidan Ryan, Sean Denyer, Kevin Kelleher, Abigail Collins

**Affiliations:** 1 Health Protection Surveillance Centre, Dublin, Ireland; 2 Department of Public Health, HSE West, Merlin Park Hospital, Galway, Ireland; 3 Department of Public Health, HSE South, St. Finbarr’s Hospital, Cork, Ireland; 4 Department of Public Health, HSE East, Dr Steeven’s Hospital, Dublin, Ireland; 5 Contact Management Programme, Health Service Executive, Ireland; 6 School of Public Health, University College Cork, Cork, Ireland; 7 Department of Public Health, HSE Mid-West, Mount Kennett House, Henry Street, Limerick, Ireland; 8 Department of Public Health, HSE Midlands, HSE Area Offices, Arden Road, Tullamore, Co. Offaly, Ireland; 9 Department of Public Health, HSE South-East, The Tower, Dublin Road, Lacken, Kilkenny, Ireland; 10 Department of Public Health, HSE North-West, Iona House, Upper Main Street, Ballyshannon, Co. Donegal, Ireland; 11 Department of Public Health, HSE North-East, Railway Street, Navan, Co. Meath, Ireland; 12 Office of Chief Clinical Officer, Public Health and Child Health HSE, Mount Kennett House, Mount Kennett Place, Henry Street, Limerick, Ireland; 13 Child Health Public Health HSE, HSE Area Offices, Arden Road, Tullamore, Co. Offaly, Ireland

**Keywords:** Epidemiology, Ireland, infection control

## Abstract

**Background:**

The role of schools in SARS-CoV-2 transmission has been a debated topic since the beginning of the COVID-19 pandemic.

**Aim:**

To examine SARS-CoV-2 transmission in all schools in Ireland during the 2020–21 school year.

**Methods:**

In a national descriptive cross-sectional study, we investigated PCR-confirmed cases of COVID-19 among students (aged < 20 years) and staff (aged ≥ 20 years) who attended school during their infectious period to identify school close contacts. SARS-CoV-2 PCR test results of all school close contacts were pooled to obtain an overall positivity rate and to stratify positivity rate by school setting and role (i.e. student or staff).

**Results:**

In total, 100,474 individuals were tested as close contacts in 1,771 schools during the 2020–21 school year. An overall close contact positivity rate of 2.4% was observed across all schools (n = 2,373 secondary cases). The highest positivity rate was seen in special schools (3.4%), followed by primary (2.5%) and post-primary schools (1.8%) (p < 0.001). Of the close contacts identified, 90.5% (n = 90,953) were students and 9.5% (n = 9,521) were staff. Overall, students had a significantly higher positivity rate than staff (2.4% vs 1.8%, p < 0.001).

**Conclusion:**

This study demonstrated that a low level of SARS-CoV-2 transmission occurred in Irish schools during the 2020-21 academic year. In the event of future pandemics, and as the COVID-19 pandemic continues, there is a need to carefully weigh up the harms and benefits associated with disrupted education to mitigate infectious disease transmission before reflexively closing classes or schools.

**Key public health message**

**What did you want to address in this study?**

During the pandemic, school closures were a frequently applied public health measure to prevent and control the spread of SARS-CoV-2, the virus causing COVID-19, but whether school attendance led to greater SARS-CoV-2 spread was a matter of debate. To add to existing knowledge and to inform public health policy, we examined COVID-19 transmission in schools in Ireland during the 2020–21 school year.

**What have we learnt from this study?**

Very few school close contacts of COVID-19 cases (less than 3%) tested positive. The school outbreaks that we detected were generally small, with 92% of outbreaks each comprising less than 10 cases.

**What are the implications of your findings for public health?**

Our findings point to limited spread of SARS-CoV-2 in schools in Ireland during the 2020–21 school year. Education is of vital importance to the health and well-being of children. School closures should be a last resort in the control of COVID-19 to avoid the consequences of reduced formal education and socialisation during children’s formative years.

## Introduction

Schools are an important source of education, physical activity and protection for children [1]. Schools not only support the development of cognitive skills in children, but also social and emotional skills, and school attendance is associated with long-term health [2]. Because of the COVID-19 pandemic, school closures occurred in over 200 countries and territories worldwide between March 2020 and February 2021, and for over 168 million children globally, schools closed for up to 1 year [1]. These measures, introduced as part of wider societal efforts to control the transmission of severe acute respiratory syndrome coronavirus 2 (SARS-CoV-2, the pathogen responsible for COVID-19) among populations, have negatively impacted children’s health and well-being in a variety of ways in the short and likely long-term [3].

The extent to which SARS-CoV-2 is spread in schools and, therefore, the extent to which school closures have been warranted remains somewhat unclear. Younger age has been associated with lower susceptibility to and lower risk of transmission of SARS-CoV-2 [4-6]. However, newer and more transmissible variants may have increased children’s susceptibility and ability to transmit SARS-CoV-2 [7-9]. Nevertheless, studies assessing the spread of SARS-CoV-2 in school settings have observed lower levels of transmission in schools compared with in the general population and other settings [10-12]. Additionally, where strict infection prevention and control (IPC) measures have been implemented, extensive transmission of SARS-CoV-2 in school settings has been largely suppressed [12,13].

For the 2020–21 school year in Ireland, specific IPC measures were recommended to minimise transmission of SARS-CoV-2 in schools (Table 1).

**Table 1:** Infection prevention and control measures implemented in schools to minimise the transmission of SARS-CoV-2, Ireland, August 2020–June 2021

Intervention	Primary schools^a^	Post-primary schools^a^
Hand hygiene	Recommended for all students and staff	Recommended for all students and staff
Physical distancing	A distance of 1 m between desks or between individual students advised, except in first 4 years of primary school (ages 5–8 years, approximately). Grouping of entire class groups as ‘class bubbles’ advised to keep a class grouping apart from other classes. Structuring of discrete groups, or ‘pods’, within class bubbles; at least 1 m distance between individual pods within the class bubble and between individuals in the pod advised.	A distance of at least 1 m (or of 2 m where possible) between individual students and staff advised. No class bubbles or pods.
Face coverings	Face coverings advised for staff, but not students.	Face coverings for staff and students advised where the 1 m distance between individuals was not achievable.
Environmental cleaning	Each setting within school advised to be cleaned at least once per day.	Each setting within school advised to be cleaned at least once per day.
Isolation for cases	Prompt identification and isolation of potentially infectious individuals advised.	Prompt identification and isolation of potentially infectious individuals advised.
Quarantine for close contacts	Exclusion of staff or student from school for 14 days from last exposure to confirmed case advised.	Exclusion of staff or student from school for 14 days from last exposure to confirmed case advised.
Vaccination	Not recommended for students during 2020–21 school year [35]. Administered to staff as part of population age-based strategy; adults aged < 65 years invited in order of decreasing age for vaccination from late April 2021 [36].	Not administered to immunocompetent students aged 12–17 years during 2020–21 school year [37]. Administered to staff as part of population age-based strategy; adults aged < 65 years invited in order of decreasing age for vaccination from late April 2021 [36].

SARS-CoV-2: severe acute respiratory syndrome coronavirus 2.

^a^ Students are approximately aged 5–12 years old in primary schools, and aged 13–18 years old in post-primary schools.

Special schools (dedicated schools in Ireland for children with special educational needs) overall followed measures similar to mainstream schools. Face coverings were not required for students, and typically, class groups operated as a ‘class bubble’, with no pod structure within bubbles. Children were aged 5–18 years in this setting.

Information in the table was adapted from [38,39].

During the 2020–21 school year, Ireland experienced two epidemiological waves of SARS-CoV-2 (Wave 2: 2 August–21 November 2020 and Wave 3: 22 November 2020–25 June 2021). For the general population, the beginning of the 2020–21 school year coincided with the publication in mid-September of the Irish Government’s ‘Plan for Living with COVID-19’ [14]. The key feature of the plan was a five-level framework of restrictions across areas such as social gatherings, events, retail, hospitality, public transport, domestic travel and others. Implementation of restrictions was intended on a regional basis depending on disease prevalence, alongside general public health measures including hand hygiene, isolation of infectious COVID-19 cases, quarantine for close contacts, mask wearing on public transport and some indoor settings, and towards the end of 2020, mass vaccination [14,15]. Because of Wave 2, the highest level of restrictions, referred to as Level 5, were introduced nationally from mid-October to early December 2020. Level 5 measures included a stay-at-home recommendation, 5 km limit for exercise, no social gatherings, no organised or sporting events, closure of leisure facilities, takeaway-only service for food and drinks premises, 25% capacity for public transport, a work-from-home recommendation except for essential workers and opening of essential retail only [16]. These measures were then eased briefly to Level 3, but quickly re-introduced in late December with Wave 3, which was characterised by a rapid increase in COVID-19 cases in November–December 2020 and subsequent dominant circulation of the Alpha (Phylogenetic Assignment of Named Global Outbreak (Pango) lineage designation B.1.1.7) variant of concern (VOC) [17]. Schools did not reopen in January 2021 because of Wave 3. Reopening of schools subsequently began on a phased basis from February 2021, with all schools open for students and staff by April 2021. For the general population, Level 5 measures continued until April 2021.

This study aimed to examine the extent of transmission of SARS-CoV-2 among students and staff in schools in the Republic of Ireland (referred to as Ireland hereafter) during the 2020–21 school year, predominantly by estimating the SARS-CoV-2 positivity rate among close contacts of infectious COVID-19 cases in schools.

## Methods

### Study design and population

This was a descriptive cross-sectional study that aimed to capture the PCR-positive SARS-CoV-2 results for all students and staff attending a school in Ireland during their infectious period. The study also captured the number of on-site school close contacts identified through contact tracing of the above confirmed cases, and close contacts’ test results.

Dates for the start and end of the school year in Ireland are not standardised, although dates for the Christmas and Easter holidays, and mid-term (October and February) breaks are. The time periods for the data collected in this study coincided with dates for each of the three terms of the 2020–21 school year, accounting for holiday periods and government-imposed restrictions during Wave 3 which prevented schools re-opening in January after the Christmas holidays for Term 2. Term 1 dated from 23 August 2020 to 22 December 2020, including October mid-term break (26–30 October 2020). Term 2 dated from 1 March 2021 for post-primary and primary schools, according to a phased re-opening with specific dates of return to school for different classes and years. The term dated from 11 February 2021 for special schools (dedicated schools in Ireland for children with special educational needs) at 50% attendance, and increased to 100% attendance from 1 March 2021, with special classes in mainstream schools permitted to re-open fully from 22 February 2021. The beginning of Term 2 was delayed because of the phased re-opening of schools in Ireland in the context of Wave 3. Term 2 ended with the Easter break (26 March–12 April 2021). Term 3 dated from 13 April 2021 to the end of June 2021 for post-primary and primary schools and to the end of May 2021 for special schools. In general, days of school were not shortened over the course of the school year, nor was there a formal national hybrid approach implemented in schools when they were re-opened.

There were 3,963 schools in Ireland during the 2020–21 school year. The population of students and staff in schools in Ireland is ca 1 million [18]. The approximate ages of students were as follows: 5–12 years in primary schools, 13–18 years in post-primary schools, 5–18 years in special schools. We defined staff as adults ≥ 20 years working at schools in any context.

### Management of COVID-19 cases and contacts

#### Case and outbreak definitions

A SARS-CoV-2 infection was confirmed by PCR testing. A COVID-19 case was defined as per the national case definition during the 2020–21 school year [19]. A COVID-19 outbreak was defined as two or more confirmed cases, or one confirmed case and one person with symptoms consistent with COVID-19 with evidence of intra-school transmission [20]. If a school outbreak of COVID-19 was declared, related cases were linked on the Computerised Infectious Disease Reporting (CIDR) system, the primary information system for surveillance and control of infectious diseases in Ireland. The declaration of a COVID-19 outbreak in a school was made by the Medical Officer of Health (MOH) in the relevant regional Department of Public Health. Directors of Public Health and Consultants in Public Health Medicine in each Department of Public Health are designated as MOHs [21]. The MOH in turn notified the national Health Protection Surveillance Centre.

#### Public health risk assessment

Public health processes were implemented in schools nationally at the beginning of the 2020–21 school year to standardise the management of cases and outbreaks of COVID-19 in these settings. If COVID-19 cases were determined to have been infectious while attending school, they were referred to the regional Department of Public Health ‘Schools Teams’ for a public health risk assessment (PHRA). Cases were considered infectious if they attended school within 48 h of symptom onset, or, if asymptomatic, within 24 h of a positive test. Once identified as positive for SARS-CoV-2, cases were advised to self-isolate for a period of days (predominantly ranging from 7–10 days; the number of days changed as public health advice evolved over the school year). 

Through the PHRA process, close contacts were identified; close contacts were identified as any person who had face-to-face contact within < 1 m of a confirmed case of COVID-19 for > 15 min in a school day or any person who had contact with a confirmed case within 1–2 m for > 15 min in a school day, with consideration given to other mitigation measures such as mask-wearing, ventilation and reported compliance with IPC measures as well as the extent of case symptoms and ability to manage those symptoms [22]. 

Priority SARS-CoV-2 testing pathways for students and staff were established. Close contacts were excluded from school for 14 days after their last exposure to a confirmed case and offered PCR testing. In the 2020 part of the school year, this was timed as soon as possible (day 0) and 7 days after last exposure (day 7), with change of timing of the second test to 10 days after exposure (day 10) in 2021. Close contacts could return to school upon receipt of a negative day 10 test result, provided they remained asymptomatic. Antigen testing was not used.

### Data sources

In Ireland, all PCR-confirmed COVID-19 cases are statutorily notified to the MOH in regional Public Health Areas; all cases are recorded on the CIDR system [23].

In March 2020, the Irish Health Service Executive (HSE) developed a national COVID-19 Contact Management Programme (CMP) to assist with contact tracing of all laboratory-confirmed COVID-19 cases. The role of this web-based information system was to notify COVID-19 cases of their test results, and to identify their close contacts. Contact tracers using the CMP telephoned cases to determine if the case attended or worked in a ‘complex setting*’* such as a school during their infectious period. If the case was in a school while infectious, the relevant regional Department of Public Health was notified by the CMP and a PHRA was undertaken by the departmental ‘Schools Team’. Cases and contacts were recorded on the CMP using a unique school identification number. Once identified, close contacts were contacted by contract tracers using the CMP, to provide appropriate public health advice and information regarding testing. For the purpose of this study, all close contacts who tested positive for SARS-CoV-2 through the schools testing pathway during the 2020–21 school year were identified as secondary cases.

Data from week 19 2021 onwards were based on SARS-CoV-2 results uploaded to the national COVID-19 Contact Management Programme. It should be noted that these data do not represent notified cases, and have not undergone the data validation procedures undertaken through the CIDR System.

### Statistical analysis

Statistical analyses were conducted using Stata version 15 (StataCorp LLC.). Categorical variables were expressed as counts and percentages. Differences between groups for categorical variables were estimated using chi-squared tests. A significance level of p < 0.05 was assumed throughout.

## Results

**Figure 1:**
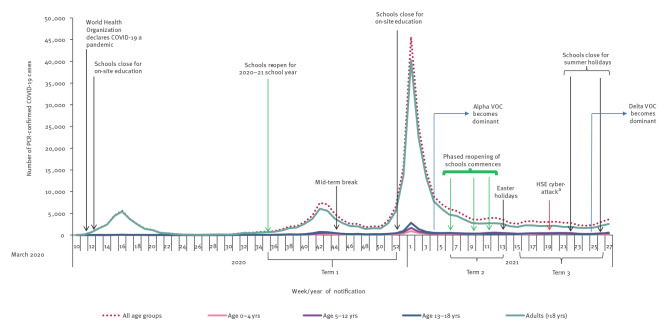
PCR-confirmed COVID-19 cases by age group in Ireland^a^, 1 March 2020–10 July 2021^b^

In total, 21,727 PCR-confirmed cases of COVID-19 in 5–18-year-olds were notified in Ireland during the three school terms of the 2020–21 school year, equating to 2.4% of the total population aged 5–18 years in Ireland (21,727/920,281) [24] and representing 18.2% of total cases of COVID-19 notified in Ireland during the dates which coincided with the 2020–21 school year (21,727/119,640). As a proportion of total cases of COVID-19 across all age groups in Ireland from March 2020 to July 2021, those aged 5–18 years comprised 13.3% (36,741/277,199) of all cases (Figure 1).

HSE: Health Service Executive; SARS-CoV-2: severe acute respiratory syndrome coronavirus 2; VOC: variant of concern.

^a^ Data from week 19 2021 onwards are based on SARS-CoV-2 results uploaded to the national COVID-19 Contact Management Programme. Please note that these data do not represent notified cases, and have not undergone the data validation procedures undertaken through the Computerised Infectious Disease Reporting System.

^b^ HSE was subject to an extensive cyber-attack on its information technology systems on 14 May 2021.

Over the three school terms of the 2020–21 school year, the proportion of all cases nationally aged 5–18 years old increased significantly, from 15.5% (9,010/58,148 total cases) in Term 1, to 17.5% (5,450/31,069 total cases) in Term 2 and 23.9% (7,267/30,423 total cases) in Term 3 (p < 0.0001, chi-squared test for trend = 957.15).

### Close contact positivity rate in schools

In total, 100,474 close contacts were tested in 1,771 schools in Ireland during the 2020–21 school year (Table 2). A total of 2,373 secondary cases were identified from school close contact testing, resulting in an overall positivity rate of 2.4%. The highest positivity rate was seen in special schools, followed by primary then post-primary schools (p < 0.001, chi-squared test = 71.3576). In Ireland, 18 schools were advised by Public Health to exclude all staff and students from school, as the whole school population had been identified as close contacts. In each of these 18 schools, all students and staff were offered testing for SARS-CoV-2, as per the management of close contacts of COVID-19 cases.

**Table 2:** SARS-CoV-2 positivity rates of school close contacts by role in all schools in Ireland, 23 August 2020–26 June 2021 (n = 100,474 close contacts)

School setting	Contacts tested	Positive^a^		Negative^a^		Unknown		p value
	n	n	%	n	%	n	%	
**Primary^b^**								
Total	73,215	1,841	2.5	71,275	97.4	99	0.1	< 0.001
Students	66,374	1,725	2.6	64,557	97.3	92	0.1	
Staff	6,841	116	1.7	6,718	98.2	7	0.1	
**Post-primary^b^**								
Total	24,603	442	1.8	24,136	98.1	25	0.1	0.88
Students	23,118	414	1.8	22,680	98.1	24	0.1	
Staff	1,485	28	1.9	1,456	98.0	1	0.1	
**Special^b^**								
Total	2,656	90	3.4	2,555	96.2	11	0.4	0.007
Students	1,461	63	4.3	1,390	95.1	8	0.5	
Staff	1,195	27	2.3	1,165	97.5	3	0.3	
**All schools **								
Total	100,474	2,373	2.4	97,966	97.5	135	0.1	0.001
Students	90,953	2,202	2.4	88,627	97.4	124	0.1	
Staff	9,521	171	1.8	9,339	98.1	11	0.1	

SARS-CoV-2: severe acute respiratory syndrome coronavirus 2.

^a^ Positive and negative results indicate that SARS-CoV-2 RNA was detected or not detected on PCR testing, respectively.

^b^ Students are ca 5–12 years old in primary schools, 13–18 years old in post-primary schools, and 5–18 years old in special schools (dedicated schools in Ireland for children with special educational needs).

Of the close contacts tested, 90.5% (n = 90,953) were students and 9.5% (n = 9,521) were staff. Overall, students had a significantly higher positivity rate than staff (2.4% vs 1.8%, p = 0.001, chi-squared test = 14.9010). On breakdown by different school settings, students had significantly higher positivity rates than staff in primary and special schools; staff, however, had a higher positivity rate in post-primary schools, but this difference was not statistically significant (1.9% vs 1.8%, p = 0.88, chi-squared test = 0.2528).

### COVID-19 outbreaks in schools

**Figure 2:**
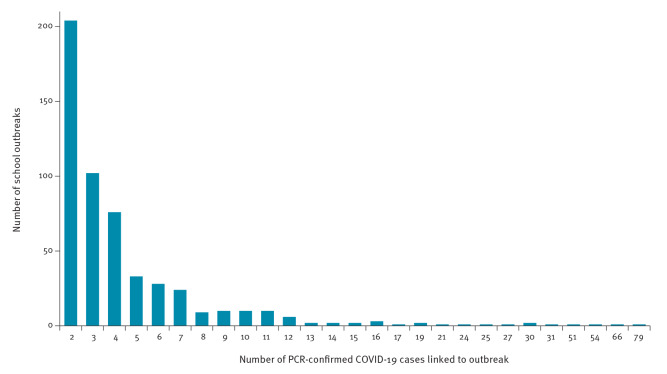
PCR-confirmed COVID-19 cases (n = 2,646) associated with reported school outbreaks (n = 601) in all schools in Ireland, August 2020–June 2021

Between August 2020–June 2021, 832 school outbreaks were notified to Public Health and recorded in the CIDR system. Overall, the 832 outbreaks totalled 3,655 cases, which included index and secondary cases (both students and staff) in schools. The size of 601 (72.2%) individual outbreaks involving 2,646 associated cases was available; these outbreaks were mainly small in size, with 34% of all outbreaks comprising two cases and 92% comprising less than 10 cases (Figure 2).

SARS-CoV-2: severe acute respiratory syndrome coronavirus 2.

### Schools with a public health risk assessment and onward testing completed

Of the 3,963 schools in Ireland, 1,771 (44.7%) reported at least one index case of COVID-19 (student or staff) who attended school during the infectious period and had school close contacts identified by a PHRA (Table 3). These data do not, therefore, include index cases who attended school while infectious but had no identified close contacts.

**Table 3:** Schools in Ireland with infectious COVID-19 index cases who had school close contacts requiring testing for SARS-CoV-2, as identified by a public health risk assessment, August 2020–June 2021 (n = 3,963 schools)

School setting^a^	Total schools in Ireland		Schools affected		Schools with 1 infectious index case requiring 1 PHRA		Schools with 2 infectious index cases requiring 2 PHRAs		Schools with 3 infectious index cases requiring 3 PHRAs		Schools with ≥ 4 infectious index cases requiring ≥ 4 PHRAs	
	n	%	n	%	n	%	n	%	n	%	n	%
Primary	3,106	100.0	1,195	38.5	499	16.1	338	10.9	159	5.1	199	6.4
Post-primary	723	100.0	499	69.0	169	23.4	129	17.8	86	11.9	115	15.9
Special	134	100.0	77	57.5	28	20.9	26	19.4	15	11.1	8	6.0
All schools	3,963	100.0	1,771	44.7	696	17.6	493	12.4	260	6.6	322	8.1

PHRA: public health risk assessment; SARS-CoV-2: severe acute respiratory syndrome coronavirus 2.

^a^ Students are ca 5–12 years old in primary schools, 13–18 years old in post-primary schools, and 5–18 years old in special schools (dedicated schools in Ireland for children with special educational needs).

## Discussion

This study demonstrated an overall positivity rate of 2.4% among identified school close contacts over the 2020–21 school year in Ireland. We also identified that school outbreaks of COVID-19 in Ireland were of small size, with few schools closed because of public health concerns during the school year. Less than half of all schools had one or more index cases who attended school during their infectious period requiring onward testing of school close contacts. Over half of schools were not affected in this way and were not subject to the potential ensuing interruption to in-person education. In total, ca 10% of the school population nationally were identified as close contacts and tested through the schools’ testing system across the three different types of school settings described [18].

The national proportion of COVID-19 cases aged 5–18 years increased over the three terms of the 2020–21 school year. This may reflect the impact of several factors, including Ireland’s COVID-19 vaccination programme, which largely progressed according to an age-based strategy for adults whereby those aged < 65 years were invited in order of decreasing age for vaccination from late April 2021 (as per Table 1). This programme was highly successful with ca 85.77% of the eligible population fully vaccinated (for their primary vaccination course) as of 16 June 2022 [25]. The high vaccination uptake likely resulted in a progressive shift in the median age of COVID-19 cases to younger unvaccinated population sub-groups during 2021 [26]. The emergence of more transmissible SARS-CoV-2 variants may also have played a role in this increase in positivity rate in the younger population. Direct comparison between the positivity rate of 2.4% among close contacts observed in this study and findings from international studies are challenging, given differences in processes for identification and testing of close contacts, uptake of testing among close contacts, use of IPC measures in schools and background community transmission of SARS-CoV-2. Nevertheless, this close contact positivity rate in schools is consistent with the international literature from Norway, Italy, Germany, Australia and the United States [11,27-30].

There was a significant difference between the close contact positivity rates across the three school settings in this study. The highest rate was seen in special schools, followed by primary and post-primary schools, respectively. This may be due to the greater educational and personal care needs of students in special schools resulting in longer time and more extensive contact with staff, and as such, greater challenges in implementing IPC measures.

We also observed a significantly lower overall positivity rate among staff identified as close contacts compared with students. There are several possible reasons for this finding. School staff may have been more effective in maintaining IPC measures, such as physical distancing, compared with children. Also, staff were required to wear face coverings in all schools, while students were required to do so in post-primary schools only. The impact of COVID-19 vaccination may also have contributed during 2021.

This study has several strengths. It was a large study, based on national data pertaining to the entire 2020–21 school year in Ireland. A single protocol was systematically followed across the country when a case of COVID-19 was notified in a school, with a detailed PHRA undertaken and close contact testing performed. The PHRA process, following a standard definition for close contacts, allowed identification of students and staff at highest risk of SARS-CoV-2 transmission following exposure to a confirmed index case, making the observed close contact positivity rate of 2.4% particularly notable. The 2020–21 school year proceeded during varying levels of background community transmission and increasing prevalence of VOCs in Ireland, specifically the Alpha variant and, to a lesser extent, Delta (Pango lineage designation B.1.617.2), in January and June 2021 respectively, thus enhancing the relevance and generalisability of our findings to other contexts.

There are some limitations to this study. Firstly, at the start of the school year, the national schools testing pathway was implemented over approximately 1 month; as such, every school might not necessarily have been included in this process initially. However, COVID-19 cases identified during most of the days of the 2020–21 school year were captured, and the impact of the selection bias is likely to have been minimal. Secondly, this study included a period of national school closures during the peak of Wave 3 of COVID-19 in Ireland. This represents a source of potential bias in our study, given the impact of community transmission of SARS-CoV-2 on school transmission [31]. Had schools been open during this period of Wave 3, it is possible that SARS-CoV-2 transmission may have been higher in schools than we observed during the rest of the school year. However, the data presented in our study are not averaged over the period of school closures during Wave 3. Also, when schools began a phased return from February 2021, Alpha was still the dominant circulating variant. Thirdly, the timing of the second PCR test for close contacts changed during the school year (day 7 post-exposure in 2020 and day 10 post-exposure in 2021). This could potentially have underestimated the close contact positivity rates, given the mean incubation period of SARS-CoV-2 [32]. Fourthly, the number of index cases considered infectious while in school was not available for this study, because the available data were aggregated. However, the number of schools where index cases with school close contacts requiring testing for SARS-CoV-2 (as identified by PHRA), was presented. Fifthly, information on compliance with IPC measures at the level of individual schools was not available for this study. This should be a priority for future research. In addition, as it was not feasible to distinguish the context of viral transmission between all cases identified in schools, it is possible that transmission linked to schools may have been over-estimated and such contact occurred through social activities or transport. Sixthly, the HSE was subject to an extensive cyber-attack on its information technology systems on 14 May 2021. The cyber-attack caused temporary disruption to the CMP system and may have resulted in some incomplete data between 14 and 29 May 2021. However, every effort was made to ensure completeness of data collated as part of the schools testing pathway during this period. As such this is likely to have had minimal impact on the quality of our data, and unlikely to have significantly biased our results. Finally, since the end of the 2020–21 school year, several new and increasingly transmissible SARS-CoV-2 VOCs have emerged – most notably, the Omicron variant [33]. Further studies are needed to understand the impact of these variants on SARS-CoV-2 transmission in school settings.

The European Centre for Disease Prevention and Control (ECDC) recommends that school closures as a measure to aid control of the COVID-19 pandemic should be viewed as a last resort, given the myriad adverse effects of such closures on the physical and mental health and well-being of school children [31]. Continued school closures have also intensified existing societal inequalities resulting in an unequal impact on more vulnerable students and their families. A consistent message in international literature and guidance is that schools are not primary drivers of SARS-CoV-2 transmission, with any potential benefits of school closures in reducing community viral transmission being far outweighed by the resulting harms to children. In this study, while the majority of identified close contacts had a negative result following PCR testing, exclusion from school resulted in substantial education time lost, which was further compounded by the mandatory universal school closures in Ireland during Wave 3 in early 2021.

## Conclusion

Our study supports international evidence which has demonstrated that educational facilities are low-risk settings for SARS-CoV-2 when there is clear focused attention to the exclusion of those with symptoms consistent with COVID-19, and appropriate IPC measures are implemented in the school setting. All children must be afforded the opportunity to continue their education as the highest priority, with minimum unnecessary interruptions. Closure of education settings must be the last resort as a control mechanism.

In the event of future pandemics, the harms and benefits associated with disrupted education to mitigate infectious disease transmission must be carefully considered before reflexively closing classes or schools. In the context of the ongoing COVID-19 pandemic, in order to maximise protection and facilitate uninterrupted education for all children, there must also be maximum uptake of COVID-19 vaccination among school staff, and students for whom vaccination is recommended, accompanied by high levels of adherence to SARS-CoV-2 mitigation measures and preparedness in educational settings.

## References

[1] United Nations Children's Fund (UNICEF). COVID-19 and School Closures: One year of education disruption. New York: UNICEF; 2021. Available from: https://data.unicef.org/resources/one-year-of-covid-19-and-school-closures/

[2] Allison MA Attisha E Council on School Health . The link between school attendance and good health. Pediatrics. 2019;143(2):e20183648. 10.1542/peds.2018-3648 30835245

[3] Viner R Russell S Saulle R Croker H Stansfield C Packer J School closures during social lockdown and mental health, health behaviors, and well-being among children and adolescents during the first COVID-19 wave. JAMA Pediatr. 2022;176(4):400-9. 10.1001/jamapediatrics.2021.5840 35040870

[4] Abrehart T Suryadinata R McCafferty C Jacobson J Ignjatovic V Robinson P Age-related differences in SARS-CoV-2 binding factors: An explanation for reduced susceptibility to severe COVID-19 among children? Paediatr Respir Rev. 2022;44:61-9. 35227628 10.1016/j.prrv.2022.01.008 PMC8823960

[5] Afonso ET Marques SM Costa LDC Fortes PM Peixoto F Bichuetti-Silva DC Secondary household transmission of SARS-CoV-2 among children and adolescents: Clinical and epidemiological aspects. Pediatr Pulmonol. 2022;57(1):162-75. 10.1002/ppul.25711 34590794 PMC8661607

[6] Bistaraki A Roussos S Tsiodras S Sypsa V . Age-dependent effects on infectivity and susceptibility to SARS-CoV-2 infection: results from nationwide contact tracing data in Greece. Infect Dis (Lond). 2022;54(3):186-95. 10.1080/23744235.2021.1995627 34743646

[7] Chen F Tian Y Zhang L Shi Y . The role of children in household transmission of COVID-19: a systematic review and meta-analysis. Int J Infect Dis. 2022;122:266-75. 10.1016/j.ijid.2022.05.016 35562045 PMC9091150

[8] Dawood FS Porucznik CA Veguilla V Stanford JB Duque J Rolfes MA Incidence rates, household infection risk, and clinical characteristics of SARS-CoV-2 infection among children and adults in Utah and New York City, New York. JAMA Pediatr. 2022;176(1):59-67. 10.1001/jamapediatrics.2021.4217 34623377 PMC8501415

[9] Fleming-Dutra KE Britton A Shang N Derado G Link-Gelles R Accorsi EK Association of prior BNT162b2 COVID-19 vaccination with symptomatic SARS-CoV-2 infection in children and adolescents during Omicron predominance. JAMA. 2022;327(22):2210-9. 10.1001/jama.2022.7493 35560036 PMC9107063

[10] Viner R Waddington C Mytton O Booy R Cruz J Ward J Transmission of SARS-CoV-2 by children and young people in households and schools: A meta-analysis of population-based and contact-tracing studies. J Infect. 2022;84(3):361-82. 10.1016/j.jinf.2021.12.026 34953911 PMC8694793

[11] Brandal LT Ofitserova TS Meijerink H Rykkvin R Lund HM Hungnes O Minimal transmission of SARS-CoV-2 from paediatric COVID-19 cases in primary schools, Norway, August to November 2020. Euro Surveill. 2021;26(1):2002011. 10.2807/1560-7917.ES.2020.26.1.2002011 33413743 PMC7791599

[12] Jordan I Fernandez de Sevilla M Fumado V Bassat Q Bonet-Carne E Fortuny C Transmission of severe acute respiratory syndrome coronavirus 2 infection among children in summer schools applying stringent control measures in Barcelona, Spain. Clin Infect Dis. 2022;74(1):66-73. 10.1093/cid/ciab227 33709138 PMC7989514

[13] Vardavas C Nikitara K Mathioudakis AG Hilton Boon M Phalkey R Leonardi-Bee J Transmission of SARS-CoV-2 in educational settings in 2020: a review. BMJ Open. 2022;12(4):e058308. 10.1136/bmjopen-2021-058308 35383084 PMC8983413

[14] National Public Health Emergency Team Policy Unit. Timeline and Detail of Public Health Restrictive Measures Advised by NPHET in Response to the COVID-19 Pandemic. Dublin: Department of Health; 2021.

[15] Department of the Taoiseach. Briefing on the government's response to COVID-19 - Wednesday 2 September 2020. Dublin: Government of Ireland; 2020. Available from: https://www.gov.ie/en/publication/5fa24-briefing-on-the-governments-response-to-covid-19-wednesday-2nd-september-2020/#introduction-and-up-to-date-public-health-guidance

[16] Department of Health. National framework for living with COVID-19. Dublin: Department of Health; 2020.

[17] Health Protection Surveillance Centre (HPSC). Summary of COVID-19 virus variants in Ireland. Dublin: HPSC. [Accessed: 2 Aug 2021]. Available from: https://www.hpsc.ie/a-z/respiratory/coronavirus/novelcoronavirus/surveillance/summaryofcovid-19virusvariantsinireland

[18] Department of Education Key Statistics. Dublin: gov.ie. [Accessed: 1 Sep 2021]. Available from: https://www.gov.ie/en/collection/key-statistics

[19] Health Protection Surveillance Centre (HPSC). COVID-19 interim case definition for Ireland. Dublin: HPSC. [Accessed: 10 Dec 2022]. Available from: https://www.hpsc.ie/a-z/respiratory/coronavirus/novelcoronavirus/casedefinitions/covid-19interimcasedefinitionforireland

[20] Health Protection Surveillance Centre (HPSC). COVID-19 outbreak case definition for Ireland. Dublin: HPSC. [Accessed: 26 May 2021]. Available from: https://www.hpsc.ie/a-z/respiratory/coronavirus/novelcoronavirus/casedefinitions/covid-19outbreakcasedefinitionforireland

[21] Health Service Executive (HSE). Medical Officer of Health. Dublin: HSE. [Accessed: 10 Dec 2022]. Available from: https://www.hse.ie/eng/services/list/5/publichealth/publichealthdepts/moh/moh.html

[22] Office of the Clinical Director for Health Protection. Educational facilities mid-term review — a focus on primary and post-primary schools. The public health perspective. Dublin: Government of Ireland; 2020. Available from: https://www.gov.ie/en/publication/0d5d2-educational-facilities-mid-term-review-a-focus-on-primary-and-post-primary-schools

[23] Health Protection Surveillance Centre (HPSC). Computerised Infectious Disease Reporting – CIDR Dublin: HPSC. [Accessed: 13 Oct 2021]. Available from: https://www.hpsc.ie/cidr

[24] Central Statistics Office (CSO). Age Groups. Dublin: CSO. [Accessed: 17 Mar 2023]. Available from: https://www.cso.ie/en/releasesandpublications/ep/p-cp3oy/cp3/agr

[25] Government of Ireland. Vaccinations. Dublin: gov.ie. [Accessed: 12 Oct 2021]. Available from: https://covid-19.geohive.ie/pages/vaccinations

[26] Health Protection Surveillance Centre (HPSC). COVID-19 14-day Epidemiology reports. Dublin: HPSC. [Accessed: 1 Sep 2021]. Available from: https://www.hpsc.ie/a-z/respiratory/coronavirus/novelcoronavirus/casesinireland/archive/covid-1914-dayepidemiologyreports2020-2021/2021/

[27] Larosa E Djuric O Cassinadri M Cilloni S Bisaccia E Vicentini M Secondary transmission of COVID-19 in preschool and school settings in northern Italy after their reopening in September 2020: a population-based study. Euro Surveill. 2020;25(49):10. 10.2807/1560-7917.ES.2020.25.49.2001911 33303065 PMC7730487

[28] Schoeps A Hoffmann D Tamm C Vollmer B Haag S Kaffenberger T Surveillance of SARS-CoV-2 transmission in educational institutions, August to December 2020, Germany. Epidemiol Infect. 2021;149:e213. 10.1017/S0950268821002077 34549699 PMC8503068

[29] Macartney K Quinn HE Pillsbury AJ Koirala A Deng L Winkler N Transmission of SARS-CoV-2 in Australian educational settings: a prospective cohort study. Lancet Child Adolesc Health. 2020;4(11):807-16. 10.1016/S2352-4642(20)30251-0 32758454 PMC7398658

[30] Zimmerman KO Akinboyo IC Brookhart MA Boutzoukas AE McGann KA Smith MJ Incidence and Secondary Transmission of SARS-CoV-2 Infections in Schools. Pediatrics. 2021;147(4):e2020048090. 10.1542/peds.2020-048090 33419869 PMC8015158

[31] European Centre for Disease Prevention and Control (ECDC). COVID-19 in children and the role of school settings in transmission - second update. Stockholm: ECDC; 2021. Available from: https://www.ecdc.europa.eu/en/publications-data/children-and-school-settings-covid-19-transmission#no-link

[32] Health Information and Quality Authority (HIQA). Evidence summary for the incubation period of COVID-19, or time to first positive test, in individuals exposed to SARS-CoV-2. Dublin: HIQA; 2021. Available from: https://www.hiqa.ie/sites/default/files/2020-11/Evidence-summary-for-the-incubation-period-of-COVID-19.pdf

[33] European Centre for Disease Prevention and Control (ECDC). SARS-CoV-2 variants of concern as of June 9 2022. Stockholm: ECDC; 2021. Available from: https://www.ecdc.europa.eu/en/covid-19/variants-concern

[34] electronic Irish Statute Book (eISB). No SI. 390/1981 - Infectious Diseases Regulations 1981. Dublin: Office of the Attorney General. [Accessed: 13 Jun 2021]. Available from: http://www.irishstatutebook.ie/eli/1981/si/390/made/en/print

[35] National Immunisation Advisory Committee. Recommendations on COVID-19 vaccination for children aged 5 to 11 years. Dublin: Royal College of Physicians of Ireland; 2021. Available from: https://rcpi-live-cdn.s3.amazonaws.com/wp-content/uploads/2021/12/NIAC-Recommendations-on-COVID-19-vaccination-for-children-aged-5-to-11-years.pdf

[36] Department of the Taoiseach. Briefing on the government's response to COVID-19 - Friday 23 April 2021. Dublin: The Government of Ireland; 2021. Available from: https://www.gov.ie/ga/foilsiuchan/f8915-briefing-on-the-governments-response-to-covid-19-friday-23rd-april2021

[37] Department of Health. Minister for Health confirms that Ireland’s COVID-19 Vaccination Programme will open to 12-15 year olds. Dublin: Government of Ireland; 2021. Available from: https://www.gov.ie/en/press-release/6b845-minister-for-health-confirms-that-irelands-covid-19-vaccination-programme-will-open-to-12-15-year-olds

[38] Department of Education and Skills. Reopening Our Schools: The Roadmap for the Full Return to School. Dublin: Government of Ireland; 2020.

[39] Department of Education and Skills. Framework to maintain Physical Distancing in the Classroom in Post Primary Schools with a Full Return of All Students for the 2020/21 School Year: Roadmap for the Full Return to School. Dublin: Department of Education and Skills; 2020. Available from: https://www.gov.ie/en/publication/d0bb9-framework-to-maintain-physical-distancing-in-the-classroom-in-post-primary-schools

